# Diagnosis and treatment of viral diseases in recipients of allogeneic hematopoietic stem cell transplantation

**DOI:** 10.1186/1756-8722-6-94

**Published:** 2013-12-17

**Authors:** Ren Lin, Qifa Liu

**Affiliations:** 1 Department of Hematology, Nanfang Hospital, Southern Medical University, Guangzhou Dadao North Street, 1838, Guangzhou China

**Keywords:** Viral infection, Allogeneic hematopoietic stem cell transplantation, Diagnosis, Treatment, Prevention

## Abstract

Viral infections are important causes of morbidity and mortality after allogeneic stem cell hematopoietic transplantation (allo-HSCT). Although most viral infections present with asymptomatic or subclinical manifestations, viruses may result in fatal complications in severe immunocompromised recipients. Reactivation of latent viruses, such as herpesviruses, is frequent during the immunosuppression that occurs with allo-HSCT. Viruses acquired from community, such as the respiratory and gastrointestinal viruses, are also important pathogens of post-transplant viral diseases. Currently, molecular diagnostic methods have replaced or supplemented traditional methods, such as viral culture and antigen detection, in diagnosis of viral infections. The utilization of polymerase chain reaction facilitates the early diagnosis. In view of lacking efficacious agents for treatment of viral diseases, prevention of viral infections is extremely valuable. Application of prophylactic strategies including preemptive therapy reduces viral infections and diseases. Adoptive cellular therapy for restoring virus-specific immunity is a promising method in the treatment of viral diseases.

## Background

Viral infections are common complications after allogeneic hematopoietic stem cell transplantation (allo-HSCT). With wide use of HLA-mismatch, unrelated and cord blood donors as alternative sources of hematopoietic stem cells, and anti-thymocyte globulin (ATG) as the standard prophylaxis of graft versus host disease (GVHD) in HLA-mismatch and unrelated donor transplantation, allo-HSCT recipients are at increasing risk for viral infections [1-5]. Fortunately, improvements in viral diagnostics, such as utilization of polymerase chain reaction (PCR)-based molecular diagnostic methods as replacement of traditional methods, facilitate the early diagnosis of viral infections [6-8]. And application of prophylactic and preemptive strategies limits the reactivation of latent viruses and development of viral diseases [9-11]. Immunotherapeutic strategies to restore virus-specific immunity, such as virus-specific cytotoxic T cells (CTL) and donor lymphocyte infusion (DLI), have been used for the treatment of viral diseases [12-14]. These developments improve outcomes of viral infections after allo-HSCT [15-17]. The aim of this article is to review the current concepts of diagnosis, prevention and treatment of viral diseases in the recipients of allo-HSCT. A brief overview will be followed by a detailed discussion on common viral diseases and viruses.

### Epidemiology

In the recipients of allo-HSCT, the difference in the reported incidence is due in part to asymptomatic or subclinical manifestations in most of viral infections and the changing epidemiology of viruses as well as differences in diagnostic methods [18-23]. Till now, large-sampled epidemiological data on overall incidence of viral infections are absent in the recipients of allo-HSCT. The limited data show that community acquired respiratory viruses (CARVs) and herpesviruses are the most common pathogens [24-26]. Among the causes of CARVs respiratory tract infections, a preponderance of respiratory syncytial virus (RSV) and parainfluenza virus (PIV) are reported, followed by influenza virus and human metapneumovirus (HMPV) [19,20,27]. In herpesvirus family, the incidence of herpes simplex virus (HSV) and varicella zoster virus (VZV) infections as well as cytomegalovirus (CMV) diseases have significantly decreased because of the effective prophylaxis [24,25]. The reports on human herpes virus (HHV)-6 diseases are increasing in allo-HSCT recipients [28-32].

### Risk factors for viral infections

In the recipients of allo-HSCT, most viral infections are opportunistic and closely related with immune status. Thus, factors influencing engraftment and immune reconstitution all potentially impact viral infections. Peripheral blood stem cell transplantation is associated with fewer viral infections than bone marrow and cord blood transplantation due to better hematopoietic and immune reconstitution [33-35]. Compared with HLA-match related transplantation, HLA-mismatch related and unrelated transplantation have an increasing risk of viral infections because immune reconstitution is delayed by the intensified GVHD prophylactic strategy, such as the use of ATG [18,36-38]. GVHD may delay immune reconstitution and is considered an independent risk factor of viral infections [36]. In addition, other factors, such as the serologic status of donors and recipients before transplantation as well as the age of recipients, may also affect the incidence of viral infections. For example, CMV-seronegative recipients receiving graft from CMV-seropositive donors are at high risk of CMV diseases [24]. Children are high-risk population of CARVs infections [26].

## Diagnostic strategies

Generally, the diagnosis of viral diseases in immunocompetent individuals is based on clinical manifestations and laboratory examination. A definitive diagnosis requires detection of specific virus in specimens obtained from involved tissues and secretions as well as blood, or even histopathologic evidences. However, such a definitive diagnosis is frequently unnecessary or unavailable due to the risk associated with the invasive procedure (e.g. infection, bleeding) [25,39]. Thus, stratified diagnosis of viral infections and diseases based on diagnostic evidences is recommended [25,39]. The clinical manifestations of viral diseases in immunocompromised individuals, including transplant recipients may be different with the immunocompetent population [40]. For instance, fever does not always occur and the disseminated diseases are more common [24,25,39]. Meanwhile, viral diseases generally occur with or subsequently to other transplant complications such as bacterial and fungal infections [24,25]. For example, CMV gastrointestinal disease is particularly difficult to diagnose because it frequently presents together with gut GVHD, and diarrhea is the same symptom of these two transplant complications [24]. Therefore, in the recipients of allo-HSCT, the diagnosis of viral diseases mainly depends on laboratory examination.

Laboratory diagnostic methods mainly include viral culture, serologic testing, antigen and nucleic acid detection [6]. Table 1 showed the common methods used in diagnosis of viral infections. The golden standard for diagnosis of most viral diseases is finding of specific histopathological features and detection of virus in the involved tissues. However, biopsy is often infeasible in allo-HSCT recipients, considering that most viral diseases occur at the early stages of transplantation with concomitant thrombocytopenia or unstable vital sign. Viral culture is unsuitable in the early diagnosis because it routinely takes days to weeks and requires specific cell lines [41]. Serologic testing requires finding of progressive increasing in antibody to identify a recent infection. Unfortunately, transplant recipients are usually unable to mount sufficient antibody because of immunosuppression. Therefore, serology is less helpful for clinical decision in the recipients of allo-HSCT [24-26]. Techniques of antigen detection, including fluorescent antibody assays and enzyme immunoassays, are rapid diagnostic methods of which results can be available within hours. But the limitation of these methods is poor sensitivity compared with molecular techniques [26,42,43]. Application of PCR technique in the detection of viral nucleic acid rapidly develops the viral diagnostics [6]. In theory, any virus can potentially be detected by PCR. Now, real-time quantitative PCR (RQ-PCR) is replacing traditional gel-based PCR because it can reflect the changes of viral loads [7,44-47]. The availability of these modern diagnostic tools facilitates early diagnosis and timely intervention of viral infections. Currently, the diagnosis of viral infections in the recipients of allo-HSCT mainly relies on PCR-based methods. To investigate new or uncommon pathogens, electron microscopy or viral culture can be used. Although biopsy has the aforementioned problems and risk, it is still required in the diagnosis of some specific viral diseases, such as EBV-associated post-transplant lymphoproliferative disorder (PTLD) [25,48].

**Table 1:** Common methods used in diagnosis of viral infections after transplantation

**Methods**	Sensitivity *	**Specificity**^**#**^	**Time**^**§**^	**Virus**	**Comments**
Culture	+++	++++	+	HSV, CMV, VZV, Influenza virus, RSV, PIV, adenovirus	Gold standard
					May take weeks before results return
Shell vial culture	++	+++	++	HSV, CMV, VZV, Influenza virus, RSV, PIV, adenovirus	Reduce the testing time compared with culture
Antigen detection	++	++++	++++	Most CARVs, herpesviruses, adenovirus	Quick results
					Poor sensitivity
PCR	++++	+++	+++	All are possible	Quick results
					High sensitivity
Histopathology and immunohistochemistry	NA	NA	NA	All are possible	Detect viruses in tissue
Electron microscopy	++	NA	++++	All are possible	Require facility and experienced staff
Serology	NA	NA	NA	NA	Less helpful in diagnosis

* “+” –“++++” indicated low sensitivity to high sensitivity; # “+” –“++++” indicated low specificity to high specificity; § “+” –“++++” indicated long testing time to short testing time. NA indicated not applicable.

## Treatment strategies

Although multiple strategies have been used [17,24-26,39], the treatment of viral diseases remains rather a challenge because few agents are available and efficacious. In the recipients of allo-HSCT, immunotherapeutic strategies to restore virus-specific immunity, such as reducing immunosuppressants, DLI and *ex vivo* generation of virus-specific CTL, are now advocated in the treatment of viral diseases [14,25,49]. However, reducing immunosuppressants is unfeasible in many patients due to potential risk of GVHD [24,25], and DLI is limited by unavailable stem cell donors and the risk of exacerbating GVHD [49]. Of note, these adoptive cellular therapies are only proven efficacious for a few viruses, such as CMV, EBV and adenovirus [12,50,51]. Early intervention has a dramatic influence upon survival and may reduce the extent of permanent injury in survivors [20,52,53]. For example, in patients with CARVs infections, treatment is more effective if started prior to development of lower respiratory tract infection (LRTI) or respiratory failure [26,54,55]. Our data showed that the patients with EBV fever without tissue involvement had better treatment response than those with end-organ diseases or PTLD [4].

## Prophylaxis strategies

Since specific therapy is limited to only several antiviral agents, prevention of viral infections is crucial to reduce the incidence and mortality of viral diseases. According to the different periods of transplantation, the strategies might be divided to prophylaxis pre-transplantation, during transplantation and post-transplantation. Before transplantation, selection of virus-seronegative stem cell donors for seronegative recipients, and decreasing virus loads in virus-seropositive donors and recipients should be considered. During transplantation, the strategies of conditioning and GVHD prophylaxis should be chosen prudently to minimize the delay of immune reconstitution. After transplantation, prophylaxis should be performed throughout the risk period such as pre-engraftment and GVHD. The incidence of HSV and VZV infections has decreased from 80% to lower than 5% in the recipients of allo-HSCT receiving antiviral prophylaxis throughout the risk period [25,56]. Preemptive therapy for reactivation of some latent viruses, such as CMV and EBV has been demonstrated to reduce the progression of viral diseases [24,25]. Vaccination, such as Measles–Mumps–Rubella and VZV vaccine, seems useful to prevent corresponding viral infections [57,58]. Influenza virus vaccine is suggested to be given to the recipients prior to each influenza season [59].

## Viral diseases after HSCT

### Respiratory diseases

Respiratory diseases after HSCT are mainly caused by CARVs [60,61]. Other viruses, such as herpesviruses and adenovirus, may also result in respiratory infections [61,62]. Majority of the patients present with upper respiratory infection, and 18-44% of these patients may progress to lower respiratory infection with mortality of 23-50% [18-21,23,63]. The incidence of respiratory diseases after allo-HSCT ranges from 3.5% to 29% [20,64], and the incidence of viral pneumonia is 2.1-14% [4,20,64-66]. Typical clinical manifestations include fever, cough, myalgias. Dyspnea is an important symptom of viral pneumonia. Some of CARVs infections show a pronounced seasonality. For example, RSV and influenza virus reach a peak incidence during the winter and spring [19]. CARVs may also result in epidemic outbreak in the wards. Herpesvirus pneumonia is usually caused by reactivation of latent viruses which occurs in severe immunosuppression such as early period of transplantation and GVHD [2,67,68].

### Encephalitis /meningitis

In immunocompetent individuals, herpesviruses are the most frequent pathogens in sporadic viral encephalitis/meningitis. A retrospective study from Schmidt-Hieber et al. showed that viral encephalitis was mainly caused by human herpes virus (HHV) -6, followed by EBV, HSV, JC virus, CMV, VZV in the recipients of allo-HSCT [69]. Our data showed that herpesvirus-associated encephalitis was mainly caused by EBV followed by HSV, CMV and VZV [70]. Recently, encephalitis caused by adenovirus is increasing [69]. The incidence of viral encephalitis after allo-HSCT was 1.2% in a retrospective study [69]. Our results revealed that the incidence of herpesviruses-associated encephalitis/meningitis was 6.3% [70]. In the recipients of allo-HSCT, viral encephalitis**/**meningitis usually occurs within 100 days post-transplantation. Clinical manifestations of viral encephalitis**/**meningitis are diverse, including fever, changes of consciousness, seizures, palsy of brain nerves and psychiatric disorders; but meningeal irritation is not common compared with healthy population [69]. The mortality of viral encephalitis**/**meningitis ranges from 0% to 80%, depending on virus types and timing of diagnosis and treatment [69,71].

### Gastroenteritis

Rotavirus and norovirus are considered main causes of viral gastroenteritis. Currently, adenovirus and CMV are increasingly recognized as important pathogens of viral gastroenteritis in the recipients of allo-HSCT. Astrovirus-associated gastroenteritis which usually occurs in children is rarely observed in the recipients of allo-HSCT [72]. Few data are available on the incidence of viral gastroenteritis after allo-HSCT. Van Kraaij et al. documented that 19% of HSCT recipients (including allo- and auto-HSCT) developed viral gastroenteritis [73]. Fortunately, the mortality caused by rotavirus and norovirus-related gastroenteritis is rare [74-77]. The most common clinical manifestation of viral gastroenteritis is diarrhea, followed by vomiting and nausea. Rotavirus and norovirus infections are seasonal with a peak incidence in winter. Most of the patients acquire the viruses from community [74,77]. The median onset time of adenovirus and CMV gastroenteritis are 60–90 days after transplantation, and usually associated with acute GVHD [76,78].

### Hepatitis

Viral hepatitis is the third cause of hepatic impairment in the recipients of allo-HSCT, and usually occurs in 3–6 months after transplantation [79]. The most frequent pathogens of viral hepatitis are hepatitis B virus (HBV) and hepatitis C virus (HCV) [80,81]. Besides, other viruses such as CMV and HSV may also result in hepatitis [24,25]. Hepatitis B and C can be caused by either virus reactivation or blood transmission. Since the carriage rates of HBV and HCV vary in different regions, the incidence of viral hepatitis varies [81,82]. Increasing virus loads in blood is valuable for diagnosis. Of note, the diagnosis of viral hepatitis should be based on exclusion of other transplant complications (e.g., sinusoidal obstruction syndrome [SOS] and GVHD). Attributed to effective prophylaxis and antiviral treatment, the mortality of hepatitis B and C is low [81,83].

### Cystitis

Post-transplant cystitis usually present with hemorrhagic cystitis (HC). According to onset time, HC is divided into early-onset HC (within 48 hours of conditioning) and late-onset HC (occurring after 48 hours). Early-onset HC is often due to the toxicity of conditioning regimen such as cyclophosphamide. It is reported that 11.6-42% of patients develop late-onset HC after transplantation [84,85]. Late-onset HC was considered to be related with reactivation of latent BK virus (BKV), but this association remains controversial [86,87]. Some studies suggested that acute GVHD might increase the risk of HC [87,88]. Recently, adenovirus and CMV were suggested to be associated with late-onset HC [88,89].

### PTLD

PTLD is a life-threatening complication following allo-HSCT. Approximately 90% of PTLD result from EBV-driven B cell proliferation poorly controlled by a weakened immune response. Recent data implicated that CMV and HHV-6 might contribute to the development of PTLD [90,91]. The incidence of PTLD varies from 0.5% to 22%, depending on the number of risk factors [3,68,92-94]. In the recipients of allo-HSCT, most of PTLD occur within 1 year post-transplantation, reaching a peak incidence within 3 months [95-97]. Isolated nodal involvement is the most common presentation. About 20% of patients present with extranodal involvement, and isolated extranodal involvement is not rare [93,96,98-100]. Clinical presentations of PTLD depend on location and the degree of organ involvement. PTLD with extranodal involvement usually have poor outcome. The mortality of PTLD is 50-64% [68,94]. Delay of diagnosis and treatment is associated with a high mortality (>90%) [96,101].

### Other viral diseases

Bone marrow suppression is common in the recipients of allo-HSCT. Graft failure is a fatal complication after transplantation. Several viruses, such as CMV, EBV, HHV-6 and adenovirus have been recognized the causes of bone marrow suppression and graft failure [24,25,39,102]. The diagnosis of virus-associated graft failure should be based on pancytopenia, bone marrow hypoplasia, detection of virus together with exclusion of GVHD, rejection and relapse [102] .

## Common viruses in allo-HSCT recipients

### Herpesviruses

The known herpesviruses resulting in human diseases include α- (HSV and VZV), β- (CMV, HHV-6,-7) and γ-herpesviruses (EBV and HHV-8). Herpesvirus infections are usually asymptomatic or subclinical in immunocompetent population. The virus becomes latent in infected cells after primary infection. When immune system is disordered or deficient, latent viruses may reactivate and result in symptomatic infections, even fatal complications [24,25]. The diagnostic methods of herpesvirus infections were summarized in Table 2.

**Table 2:** Laboratory diagnosis of herpesviruses

**Viruses**	**Methods**
HSV	PCR(preferred); antigen detection [25]
VZV	PCR(preferred); Immunofluorescent-antibody staining [25]
CMV	PCR(preferred); CMV antigen (pp65) detection [24,103]
EBV	PCR; immunohistochemistry or in situ hybridization to detect EBV in biopsy specimens [25]
HHV6-8	PCR [24]

#### HSV

Up to 80% of the healthy population have history of HSV-1 and −2 infections [25]. HSV-1 infection is more common than HSV-2. After primary infection, the virus becomes latent in the neuronal cells. Historically, 80% of HSV-seropositive patient developed reactivation after allo-HSCT without antiviral prophylaxis [104]. Fortunately, the incidence of HSV reactivation has now decreased to 0-3% because of prophylactic acyclovir [56,105]. HSV causes a spectrum of diseases, such as herpes, oesophagitis, bone marrow suppression, respiratory tract diseases, hepatitis and encephalitis in the recipients of allo-HSCT [25]. Due to the high rate of HSV reactivation, prophylactic oral acyclovir has been administered routinely in allo-HSCT recipients. Intravenous acyclovir should be considered for patients with poor drug absorption [17,106]. Valacyclovir is an alternative prophylactic agent with good bioavailability [107-109]. Acyclovir is recommended as the therapy for severe mucocutaneous or visceral HSV disease in transplant recipients [25]. Valaciclovir and famciclovir are considered as alternatives for less serious manifestations of HSV diseases [25]. The recommended drug for acyclovir-resistant HSV is foscarnet [17]. Cidofovir might be effective to treat HSV infection which is resistant to both acyclovir and foscarnet [110].

#### VZV

Varicella caused by primary VZV infection is a common childhood disease. After primary infection, VZV establishes latency in the dorsal root ganglia in immunocompetent host. The reactivation of VZV results in herpes zoster [111,112]. In the recipients of allo-HSCT, VZV is also an important cause of viral encephalitis. VZV immunization is advocated in recipients without a history of varicella. Varicella vaccine has been showed to be safe in children with leukemia, but few data are available in transplant recipients [106]. Besides, vaccination of VZV-seronegative individuals who may be in contact with the patients during transplantation should be done [25]. Zoster immune globulin (ZIG) and varicella-zoster immune globulin (VZIG) are passive antibody prophylaxis in seronegative recipients after exposure to varicella [113]. Acyclovir and valacyclovir prophylaxis were proven effective in several trials [9,114-117]. Antiviral therapy with acyclovir is recommended in the treatment of VZV infection [25]. Acyclovir has been shown to reduce the progression and dissemination of VZV infection [118,119]. Treatment with brivudin or famiciclovir is effective in immunocompromised population [120,121]. Foscarnet and cidofovir are alternative agents against acyclovir-resistant VZV infection [122].

#### CMV

CMV infects 70-80% of the healthy individuals and establishes latency in peripheral blood monocytes and tissue macrophages. Till now, CMV remains one of the most important viruses and causes of death in the recipients of allo-HSCT. CMV-associated end-organ diseases include pneumonia, enteritis, hepatitis, retinitis and encephalitis, and so on; CMV syndrome is defined as CMV-associated fever without sign of CMV end-organ disease [24]. Majority of CMV infections are caused by reactivation of virus which usually occurs within 3 months post-transplantation [8,24,78]. Approximately 75% of CMV-seropositive recipients develop CMV reactivation, and 20-30% of these patients develop CMV disease without intervention [17,24]. Preemptive therapy based on CMV antigenemia or DNA-emia significantly reduces the development of CMV disease in allo-HSCT patients [24,123]. However, the mortality of CMV disease is more than 50% even with treatments [67,124]. The diagnosis of CMV infection includes CMV viremia, CMV syndrome and CMV end-organ disease [24]. Historically, CMV antigen (pp65) detection was widely used in diagnosis of CMV infection. Recently, PCR is replacing antigenemia assay to be the preferred diagnostic method due to higher sensitivity [24,103].

Preemptive therapy based on CMV viremia has become the standard prevention of CMV diseases after transplantation [24]. The first-line preemptive therapy is ganciclovir with a minimum duration of 2 weeks depending on whether CMV is detected at the end of the course [24,125,126]. The main side effect of ganciclovir is bone marrow suppression which results in the increase of bacterial and fungal infection [127,128]. Valganciclovir is an alternative with good bioavailability [129-131]. Foscarnet and cidofovir are the second-line prophylactic agents considering of drug-associated toxicity [24].

Ganciclovir is the first-line treatment of CMV diseases. The recommended therapy of CMV pneumonia is a combination of intravenous ganciclovir and high dose immune globulin [24,124,132]. In view of toxicity and effective rate, cidofovir and foscarnet are used as second-line therapy of CMV diseases [24]. Ganciclovir resistance is uncommon and usually mediated through mutations in the UL97 gene. Cidofovir is used in the treatment of CMV disease which is resistant to ganciclovir and foscarnet, with an effective rate of 50% [133]. Since it has been known that specific immune response to CMV is important to control reactivation, CMV-specific CTL has been used in prophylaxis and treatment of CMV viremia in several studies [50,51,134]. Leen et al. reported that the response rate of CMV-specific CTL was 73.9% in the treatment of CMV diseases after allo-HSCT [50].

#### EBV

Approximately 90% of healthy adults have been infected by EBV. After primary infection, EBV is latent in B cells (6, 8). Primary EBV infection or reactivation usually induces asymptomatic infection or infectious mononucleosis in immunocompetent people. However, EBV results in a spectrum of diseases in the recipients of allo-HSCT, ranging from fever, end-organ disease (pneumonia, encephalitis/myelitis, and hepatitis) to PTLD [4]. Among these diseases, PTLD is most common [4,25]. Our data showed that the 3-year cumulative incidence of EBV disease were 15.6% in the recipients of allo-HSCT, with the PTLD incidence of 9.9% [4]. EBV disease is usually caused by reactivation of latent virus after allo-HSCT. After transplantation, 14-65% of recipients developed EBV reactivation, depending on different risk factors that the recipients have [37,68,135]. The diagnosis of EBV infection includes EBV viremia, probable end-organ disease and proven end-organ disease as well as PTLD [25].

Since rapid increase of EBV-DNA loads in blood is considered to be related with subsequent EBV diseases [136], routine monitoring of EBV viral loads is necessary after transplantation [25]. Preemptive therapy based on EBV-DNA loads in blood and risk factors for EBV disease has yielded good results [25]. Preemptive therapy is now developing mainly in two directions: adoptive cellular therapy (EBV-specific CTL) and B-cell depletion with monoclonal antibodies. EBV-specific CTL has been demonstrated effective to prevent EBV disease in several studies [135,137], but the production of CTL requires time. Besides, reduction of immunosuppressants is an ideal preemptive therapy, but frequently not available due to the risk of GVHD. Rituximab is easily available and has shown little toxicity [138]. Based on the above, rituximab is recommended as the preferred preemptive therapy, followed by reduction of immunosuppresants and EBV-specific CTL in the European guidelines [25].

The therapeutic strategies of EBV disease include anti-virus, restoration of T cell response and clearance of the EBV infected cells. Antiviral agents (e.g. acyclovir and ganciclovir) can reduce EBV replication, but is not active in PTLD presumably because that viral thymidine kinase expression is low during lytic phase and lack during latency [139,140]. Recently, a novel agent arginine butyrate, which induces EBV thymidine kinase transcription, has been shown *in vitro* to render latently infected EBV-immortalized B cells susceptible to ganciclovir [139,140]. Treatments to restore T-cell reactivity include reduction of immunosuppressants and adoptive cellular therapy (CTL and DLI) [25]. Anti-CD20 monoclonal antibody (rituximab) is used to clear EBV-infected B cells [25].

According to the European guidelines, rituximab is a recommendation of highest priority for treatment of PTLD; other first-line treatment includes reducing immunosuppressants, EBV-CTL and DLI [25]. Chemotherapy is recommended as the second-line therapy [25]. The response rate of rituximab monotherapy was reported 44-69% whereas the relapse rate was 18-32% [141-144]. Compared with rituximab, adoptive cellular immunotherapy has higher response rate (50-88%) and fewer relapse (0%) [12,145,146]. Nevertheless, the utilization of adoptive cellular therapy is limited by the aforementioned disadvantages such as time and facilities required by CTL production as well as potential risk of GVHD caused by DLI [12,14,97,137,145]. Chemotherapy is reported to induce remissions in 40-50% of the PTLD patients but with significant treatment-related mortality and relapse rate [141,147]. Therefore, in the ‘era of rituximab’, chemotherapy is barely used unless for CD20-negative PTLD or combination with rituximab [145]. To date, there are no randomized trials to compare the efficacy between rituximab alone and rituximab combined with chemotherapy. Trappe et al. [148] suggested that sequential first-line treatment with rituximab followed by chemotherapy is more efficacious than first-line rituximab monotherapy followed by chemotherapy at progression or relapse. In our study, we introduced a sequential therapeutic strategy that is rituximab-based treatments followed by adoptive cellular immunotherapy. The results revealed that this strategy might elevate response rate and decrease the relapse rate. Besides, this strategy might overcome the drawback of long time frame to product EBV-CTL and reduce the risk of GVHD caused by DLI. It remains a matter of discussion that whether histology subtypes of PTLD affect the outcome of rituximab-based treatments [148,149]. The prognosis of PTLD with extranodal or multi-organ involvement is dismal compared with isolated nodal involvement [34,150]. Some studies suggested that intrathecal rituximab is efficacious against PTLD with CNS involvement [151-153]. Other therapeutic options include local radiotherapy and operation, which is mainly for the patients with significant compression symptoms [145].

Clinical data on EBV fever and end-organ diseases are quite limited. Rituximab seems efficacious to treat EBV fever, with a response rate of 100% [4,154]. The treatment strategies of EBV end-organ diseases are similar with PTLD. However, the efficacy of rituximab monotherapy in patients with end-organ diseases seemed poorer than those with PTLD [4].

#### HHV6-8

Approximately 50% of recipients develop HHV-6 reactivation after transplantation [28,30]. HHV-6 diseases include encephalitis, interstitial lung disease and delayed engraftment [24,31]. Two small-sampled studies suggested that ganciclovir might be effective to prevent HHV-6 reactivation in allo-HSCT recipients [155,156]. However, no widely accepted prophylactic strategy is recommended considering of the drug toxicity and the low incidence of HHV-6 diseases [24]. Both ganciclovir and foscarnet were reported to be effective against HHV-6 diseases [157].

HHV-7 infection/reactivation is infrequent in the recipients of allo-HSCT, and little information about HHV-7 diseases is available. Therefore, prophylaxis and treatment of HHV-7 infection remain unclear [24].

HHV-8 is recognized the cause of Kaposi’s sarcoma in human immunodeficiency virus (HIV) infected patients [158]. HHV-8 infection is rare in the recipients of allo-HSCT, and usually results in non-malignant diseases such as hepatitis, bone marrow suppression [159,160]. Currently, clinical data on prevention and treatment of HHV-8 diseases after allo-HSCT are based on case reports. Cessation of immunosuppressants and foscarnet were used for treatment, and the efficacy requires further study [161,162].

### CARVs

CARVs, including orthymyxo- (influenza virus), paramyxo- (RSV, PIV, HMPV), picorna- (human rhinovirus [HRhV]), coronaviruses (HCoVs), human bocavirus (HBoV), and polyomaviruses, are important pathogens of respiratory tract infections in the recipients of allo-HSCT, [26]. Significant overlap in clinical manifestations is observed in CARVs infections, and atypical presentations are common. Table 3 summarized the diagnostic methods and epidemiology of CARVs infections in the recipients of HSCT. Antigen detection has a good specificity and a short turn-around time of several hours, but a lower sensitivity compared with PCR [59]. Currently, PCR for detection of virus nucleic acid is preferred in diagnosis [26].

**Table 3:** CARVs infections after HSCT

**Viruses**	**Diagnostic methods**	**Incidence of infection**	**Progression to LRTI**	**Mortality with LRTI**
RSV	PCR(preferred); Antigen detection; culture	2.2-5.8% [27,64,163]	17-84% [19,164]	7-83% [19,164]
PIV	PCR(preferred); Antigen detection; culture	Up to 17.9% [19,165]	50%[19,165]	Up to 75% [166]
Influenza virus	PCR(preferred); Antigen detection; culture	1.7-9.0% [19,21,23]	18-44% [19,21,23]	5-37% [19,21,23]
HMPV	PCR(preferred); Antigen detection;	2.5-9% [26]	21-40%	33-40%
HRhV	PCR(preferred); culture	22.3% [26]	<10% [26]	?
HCoV	PCR	11% [167]	?	?
HBoV	PCR	2.1%[167]	?	?

?, absence of significant studies.

Infection control is the mainstay of prevention against CARVs disease. Healthcare facilities and infection control measures, including isolation and strict protection measures for healthcare workers and contacts, should be applied to HSCT recipient [26,168]. Besides, recipients and contacts should adhere to good personal hygiene. Recipients should avoid contact with individuals with CARVs respiratory infections in the hospital and the community [26,168].

#### RSV

RSV is one of the most common respiratory pathogens in most series including HSCT recipients. Limited data are available on prevention of RSV infection. In some studies, treating upper respiratory tract disease was effective to reduce the progression to pneumonia and improve the outcome [26,163,164]. But some other reports did not confirm this result [169,170]. Aerosolized ribavirin with or without RSV-specific immunoglobulin or intravenous immunoglobulin has been used to treat RSV pneumonia, with the 30-days survival of approximately 60% [19,54,64,163,171]. Treatment started before respiratory failure is associated with improved outcome [19,54,64]. Intravenous ribavirin is less effective but increases the side effects [55]. The efficacy of palivizumab (an RSV-specific monoclonal antibody) in HSCT recipients is not well defined [172,173].

#### PIV

PIV infection occurs throughout the year, with the highest incidence in July and September [166]. The prophylactic and therapeutic strategies against PIV infection remain a matter of discussion. A large-sampled retrospective analysis suggested that aerosolized ribavirin with or without intravenous immunoglobulin did not improve the outcome of PIV pneumonia [165]. Elizaga et al. documented that the effective rate of aerosolized ribavirin therapy was 100% in the treatment of upper tract infections, whereas only 25% of patients with PIV pneumonia survived [166].

#### Influenza virus

To prevent influenza after transplantation, the European guidelines recommended that vaccination should be performed in HSCT recipients with seasonal influenza vaccine [59]. Meanwhile, vaccination of healthcare workers and close contacts with HSCT recipients is advocated [59]. Prophylaxis with oseltamivir seems useful after exposure and during the period of influenza circulation [59,174]. M2 inhibitors, amantadine and rimantidine have no longer been used for treatment due to widespread resistance [175,176]. Neuraminidase inhibitors (oral oseltamivir or inhalational zanamivir) are now the most widely used therapeutic agents for influenza, with an effective rate of 46-100% [21,177,178]. Intervention initiated within 48 hours of symptom onset was associated with reduced risk both for pneumonia and the need for intensive care [21,179].

#### Other CARVs

At present, respiratory infections after HSCT caused by HCoV, HMPV and HRhV have been increasingly recognized. In our recent study, HCoV was found in 16.2% of the recipients within 6 months after HSCT, including 12% of LRTI; HMPV and HRhV was found in 5.4% and 2.7% of the recipients, respectively. The efficacious prevention and treatment of HMPV infections have not been well described. Ribavirin and/or intravenous immunogloblin were used to treat HMPV pneumonia in several studies but the efficacy was not defined [26,180]. The prevention and treatment strategies of HRhV are limited by the lack of antiviral agents and clinical trials. There are no recommendations on prophylaxis and treatment due to absence of effective antiviral agents and appropriate clinical studies [26].

### Adenovirus

Adenovirus is increasingly recognized as an important pathogen in immunocompromised individuals, especially in the recipients of allo-HSCT. Adenovirus infection may arise from either reactivation of latent virus or acquisition from community. Contrary to self-limited infection in most immunocompetent individuals, adenovirus causes lethal end-organ or systemic diseases in immunocompromised patients [39]. The incidence of adenovirus infection ranges from 0-6% in adult allo-HSCT recipients, and half of these patients developed adenovirus-associated diseases [18,22]. Adenovirus diseases include respiratory tract disease, gastroenteritis, encephalitis, myocarditis, nephritis and multiple organ involvement [18,22]. The mortality was reported as high as 100% in adenovirus pneumonia [39] and 61% in disseminated disease [181]. Now, PCR is the standard diagnostic method of adenovirus infection [39]. Other diagnostic approaches include viral culture and antigen detection [39].

Strict isolation and hygiene measures are advocated in patients shedding the adenovirus to prevent horizontal transmission and nosocomial outbreaks. Bordigoni et al. [182] suggested cidofovir or DLI seemed encouraging approaches to prevent adenovirus diseases, whereas ribavirin and vidarabin were ineffective.

The efficacy of ribavirin is controversial in the treatment of adenovirus diseases [182-184]. Several studies documented that cidofovir was efficacious against adenovirus [75,76,182,185]. Considering of the insufficient immune response to control adenovirus reactivation in the patients, adenovirus-specific CTL seems a promising therapy [186,187]. Leen et al. reported that the effective rate of adenovirus-specific CTL was 77.8% in the recipients of allo-HSCT [50]. In addition, reduction of immunosuppressants is recommended for prophylaxis and treatment if possible [18].

### Gastroenteritis viruses

Rotavirus and norovirus are important pathogens of viral gastroenteritis. The incidence of rotavirus infection is 6.7-11.5% in HSCT recipients, and death is rare [73,74]. Antigen detection as well as PCR is used in diagnosis of rotavirus infection. At present, norovirus as a cause of gastroenteritis after allo-HSCT is not well recognized [188]. In pediatric HSCT recipients, the incidence of norovirus-associated gastroenteritis was reported 12.9% and no norovirus-associated mortality was observed [77]. Detection of norovirus RNA by PCR is the main diagnostic evidence. Till now, little information is available about prevention and treatment of rotavirus and norovirus in the HSCT population. Oral immunoglobulin and nitazoxanide have been used in some studies [74].

### Hepatitis viruses

Among hepatitis viruses, HBV and HCV are important causes of hepatic impairment in the recipients of allo-HSCT. Hepatitis virus-positive donor should be avoided if alternatives exist.

For HBV-negative recipients, pre-transplant vaccination and HBV-specific immune globulin should be considered if the donors are HBV surface antigen positive. For HBV-positive recipients, administration of antiviral agents (i.e. lamivudine, famciclovir, Entecavir and adefovir) pre- and post-transplantation is advocated to reduce HBV replication [189,190].

Administration of ribavirin and interferon should be considered to decrease or clear the viral loads before transplantation in donors and recipients who are HCV positive [17,191]. Interferon with or without ribavirin is used for treatment of hepatitis C [192,193].

### Polyomaviruses

JC virus (JVC) which belongs to polyomavirus family is known a pathogen of progressive multifocal leukoencephalopathy (PML) in patients with HIV infection. Reports of PML are quite rare in the recipients of allo-HSCT [194]. Currently, treatment and prevention of JCV in HSCT recipients remain unclear. Treatments consisting of reduction of immunosuppressants, cidofovir and serotonin-reuptake inhibitor as well as of JCV-specific CTL infusion were demonstrated to produce a favorable clinical outcome [194].

As mentioned, BKV as a member of polyomavirus family is one of the causes of HC in allo-HSCT recipients. It is reported that ciprofloxacin and other fluoroquinolones may be useful to prevent BKV reactivation [195]. Cidofovir has been used in the treatment of BKV-associated HC [196,197].

## Conclusion

Viral diseases are important complications after HSCT. Development of viral diagnostics improves the early diagnosis and increases the diagnostic rate of some newly discovered or uncommon viruses in allo-HSCT recipients. Based on the pathogenesis of viral diseases, prophylaxis, especially preemptive therapy can limit reactivation of some latent viruses. Immunotherapeutic strategies to restore virus-specific immunity are attractive methods in the treatment of viral diseases.

## Competing interests

The authors declare that they have no competing interests.

## Authors’ contributions

The manuscript is derived from literature summarizing reports prepared by RL and QFL. Both authors wrote the manuscript and have read and approved the final version.

## References

[B1] Gratwohl A Brand R Frassoni F Rocha V Niederwieser D Reusser P Einsele H Cordonnier C Cause of death after allogeneic haematopoietic stem cell transplantation (HSCT) in early leukaemias: an EBMT analysis of lethal infectious complications and changes over calendar time Bone Marrow Transplant 2005 6 9 757 769 10.1038/sj.bmt.1705140 16151426

[B2] Ozdemir E Saliba RM Champlin RE Couriel DR Giralt SA de Lima M Khouri IF Hosing C Kornblau SM Anderlini P Risk factors associated with late cytomegalovirus reactivation after allogeneic stem cell transplantation for hematological malignancies Bone Marrow Transplant 2007 6 2 125 136 10.1038/sj.bmt.1705699 17530009

[B3] Hale G Waldmann H Risks of developing Epstein-Barr virus-related lymphoproliferative disorders after T-cell-depleted marrow transplants. CAMPATH Users Blood 1998 6 8 3079 3083 9531622

[B4] Xuan L Jiang X Sun J Zhang Y Huang F Fan Z Guo X Dai M Liu C Yu G Spectrum of Epstein-Barr virus-associated diseases in recipients of allogeneic hematopoietic stem cell transplantation Transplantation 2013 6 6 560 566 10.1097/TP.0b013e31829d38af 23842192

[B5] Lv M Huang XJ Allogeneic hematopoietic stem cell transplantation in China: where we are and where to go J Hematol Oncol 2012 6 10 10.1186/1756-8722-5-10 22424172 PMC3353833

[B6] Storch GA Diagnostic virology Clin Infect Dis 2000 6 3 739 751 10.1086/314015 11017824

[B7] Watzinger F Ebner K Lion T Detection and monitoring of virus infections by real-time PCR Mol Aspects Med 2006 6 2–3 254 298 16481036 10.1016/j.mam.2005.12.001 PMC7112306

[B8] Sahin DG Gunduz E Kasifoglu N Akay OM Us T Gulbas Z Cytomegalovirus DNAemia detected with real-time polymerase chain reaction in hematopoietic stem cell transplant patients Adv Ther 2013 6 8 784 791 10.1007/s12325-013-0049-9 23959787

[B9] Klein A Miller KB Sprague K DesJardin JA Snydman DR A randomized, double-blind, placebo-controlled trial of valacyclovir prophylaxis to prevent zoster recurrence from months 4 to 24 after BMT Bone Marrow Transplant 2011 6 2 294 299 10.1038/bmt.2010.99 20421867

[B10] Goodrich JM Mori M Gleaves CA Du Mond C Cays M Ebeling DF Buhles WC DeArmond B Meyers JD Early treatment with ganciclovir to prevent cytomegalovirus disease after allogeneic bone marrow transplantation N Engl J Med 1991 6 23 1601 1607 10.1056/NEJM199112053252303 1658652

[B11] Green ML Leisenring W Stachel D Pergam SA Sandmaier BM Wald A Corey L Boeckh M Efficacy of a viral load-based, risk-adapted, preemptive treatment strategy for prevention of cytomegalovirus disease after hematopoietic cell transplantation Biol Blood Marrow Transplant 2012 6 11 1687 1699 10.1016/j.bbmt.2012.05.015 22683614 PMC3467354

[B12] Doubrovina E Oflaz-Sozmen B Prockop SE Kernan NA Abramson S Teruya-Feldstein J Hedvat C Chou JF Heller G Barker JN Adoptive immunotherapy with unselected or EBV-specific T cells for biopsy-proven EBV+ lymphomas after allogeneic hematopoietic cell transplantation Blood 2012 6 11 2644 2656 10.1182/blood-2011-08-371971 22138512 PMC3311278

[B13] Mackinnon S Thomson K Verfuerth S Peggs K Lowdell M Adoptive cellular therapy for cytomegalovirus infection following allogeneic stem cell transplantation using virus-specific T cells Blood Cells Mol Dis 2008 6 1 63 67 10.1016/j.bcmd.2007.07.003 17869548

[B14] Papadopoulos EB Ladanyi M Emanuel D Mackinnon S Boulad F Carabasi MH Castro-Malaspina H Childs BH Gillio AP Small TN Infusions of donor leukocytes to treat Epstein-Barr virus-associated lymphoproliferative disorders after allogeneic bone marrow transplantation N Engl J Med 1994 6 17 1185 1191 10.1056/NEJM199404283301703 8093146

[B15] Ali N Adil SN Shaikh MU Moosajee M Masood N Outcome of match related allogeneic stem cell transplantation procedures performed from 2004 till 2011 Exp Hematol Oncol 2012 6 1 13 10.1186/2162-3619-1-13 23210643 PMC3514083

[B16] Loren AW Porter DL Stadtmauer EA Tsai DE Post-transplant lymphoproliferative disorder: a review Bone Marrow Transplant 2003 6 3 145 155 10.1038/sj.bmt.1703806 12621474

[B17] Ljungman P Prevention and treatment of viral infections in stem cell transplant recipients Br J Haematol 2002 6 1 44 57 10.1046/j.1365-2141.2002.03515.x 12100127

[B18] Chakrabarti S Mautner V Osman H Collingham KE Fegan CD Klapper PE Moss PA Milligan DW Adenovirus infections following allogeneic stem cell transplantation: incidence and outcome in relation to graft manipulation, immunosuppression, and immune recovery Blood 2002 6 5 1619 1627 10.1182/blood-2002-02-0377 12176880

[B19] Nichols WG Gooley T Boeckh M Community-acquired respiratory syncytial virus and parainfluenza virus infections after hematopoietic stem cell transplantation: the Fred Hutchinson Cancer Research Center experience Biol Blood Marrow Transplant 2001 6 Suppl 11S 15S 11777098 10.1053/bbmt.2001.v7.pm11777098

[B20] Martino R Porras RP Rabella N Williams JV Ramila E Margall N Labeaga R Crowe JJ Coll P Sierra J Prospective study of the incidence, clinical features, and outcome of symptomatic upper and lower respiratory tract infections by respiratory viruses in adult recipients of hematopoietic stem cell transplants for hematologic malignancies Biol Blood Marrow Transplant 2005 6 10 781 796 10.1016/j.bbmt.2005.07.007 16182179 PMC3347977

[B21] Nichols WG Guthrie KA Corey L Boeckh M Influenza infections after hematopoietic stem cell transplantation: risk factors, mortality, and the effect of antiviral therapy Clin Infect Dis 2004 6 9 1300 1306 10.1086/425004 15494906

[B22] Flomenberg P Babbitt J Drobyski WR Ash RC Carrigan DR Sedmak GV McAuliffe T Camitta B Horowitz MM Bunin N Increasing incidence of adenovirus disease in bone marrow transplant recipients J Infect Dis 1994 6 4 775 781 10.1093/infdis/169.4.775 8133091

[B23] Lewis VA Champlin R Englund J Couch R Goodrich JM Rolston K Przepiorka D Mirza NQ Yousuf HM Luna M Respiratory disease due to parainfluenza virus in adult bone marrow transplant recipients Clin Infect Dis 1996 6 5 1033 1037 10.1093/clinids/23.5.1033 8922798

[B24] Ljungman P de la Camara R Cordonnier C Einsele H Engelhard D Reusser P Styczynski J Ward K Management of CMV, HHV-6, HHV-7 and Kaposi-sarcoma herpesvirus (HHV-8) infections in patients with hematological malignancies and after SCT Bone Marrow Transplant 2008 6 4 227 240 10.1038/bmt.2008.162 18587440

[B25] Styczynski J Reusser P Einsele H de la Camara R Cordonnier C Ward KN Ljungman P Engelhard D Management of HSV, VZV and EBV infections in patients with hematological malignancies and after SCT: guidelines from the Second European Conference on Infections in Leukemia Bone Marrow Transplant 2009 6 10 757 770 10.1038/bmt.2008.386 19043458

[B26] Hirsch HH Martino R Ward KN Boeckh M Einsele H Ljungman P Fourth European Conference on Infections in Leukaemia (ECIL-4): guidelines for diagnosis and treatment of human respiratory syncytial virus, parainfluenza virus, metapneumovirus, rhinovirus, and coronavirus Clin Infect Dis 2013 6 2 258 266 10.1093/cid/cis844 23024295 PMC3526251

[B27] Peck AJ Englund JA Kuypers J Guthrie KA Corey L Morrow R Hackman RC Cent A Boeckh M Respiratory virus infection among hematopoietic cell transplant recipients: evidence for asymptomatic parainfluenza virus infection Blood 2007 6 5 1681 1688 10.1182/blood-2006-12-060343 17502457 PMC1975849

[B28] Kadakia MP Rybka WB Stewart JA Patton JL Stamey FR Elsawy M Pellett PE Armstrong JA Human herpesvirus 6: infection and disease following autologous and allogeneic bone marrow transplantation Blood 1996 6 12 5341 5354 8652850

[B29] Dewhurst S Human herpesvirus type 6 and human herpesvirus type 7 infections of the central nervous system Herpes 2004 6 Suppl 2 105A 111A 15319097

[B30] Yoshikawa T Asano Y Ihira M Suzuki K Ohashi M Suga S Kudo K Horibe K Kojima S Kato K Human herpesvirus 6 viremia in bone marrow transplant recipients: clinical features and risk factors J Infect Dis 2002 6 7 847 853 10.1086/339411 11920307

[B31] Jeulin H Agrinier N Guery M Salmon A Clement L Bordigoni P Venard V Human herpesvirus 6 infection after allogeneic stem cell transplantation: incidence, outcome, and factors associated with HHV-6 reactivation Transplantation 2013 6 10 1292 1298 10.1097/TP.0b013e318289958b 23514960

[B32] Ljungman P Singh N Human herpesvirus-6 infection in solid organ and stem cell transplant recipients J Clin Virol 2006 6 Suppl 1 S87 S91 17276376 10.1016/S1386-6532(06)70018-X

[B33] Storek J Dawson MA Storer B Stevens-Ayers T Maloney DG Marr KA Witherspoon RP Bensinger W Flowers ME Martin P Immune reconstitution after allogeneic marrow transplantation compared with blood stem cell transplantation Blood 2001 6 11 3380 3389 10.1182/blood.V97.11.3380 11369627

[B34] Styczynski J Gil L Tridello G Ljungman P Donnelly JP van der Velden W Omar H Martino R Halkes C Faraci M Response to rituximab-based therapy and risk factor analysis in epstein barr virus-related lymphoproliferative disorder after hematopoietic stem cell transplant in children and adults: a study from the infectious diseases working party of the European group for blood and marrow transplantation Clin Infect Dis 2013 6 6 794 802 10.1093/cid/cit391 23771985

[B35] McGoldrick SM Bleakley ME Guerrero A Turtle CJ Yamamoto TN Pereira SE Delaney CS Riddell SR Cytomegalovirus-specific T cells are primed early after cord blood transplant but fail to control virus in vivo Blood 2013 6 14 2796 2803 10.1182/blood-2012-09-453720 23412093 PMC3617639

[B36] Chakrabarti S Mackinnon S Chopra R Kottaridis PD Peggs K O'Gorman P Chakraverty R Marshall T Osman H Mahendra P High incidence of cytomegalovirus infection after nonmyeloablative stem cell transplantation: potential role of Campath-1H in delaying immune reconstitution Blood 2002 6 12 4357 4363 10.1182/blood.V99.12.4357 12036862

[B37] Xuan L Huang F Fan Z Zhou H Zhang X Yu G Zhang Y Liu C Sun J Liu Q Effects of intensified conditioning on Epstein-Barr virus and cytomegalovirus infections in allogeneic hematopoietic stem cell transplantation for hematological malignancies J Hematol Oncol 2012 6 46 10.1186/1756-8722-5-46 22856463 PMC3422173

[B38] Huan C Kai-yan L Lan-ping X Dai-hong L Yu-hong C Xiang-yu Z Wei H Xiao-hui Z Yu W Yuan-yuan Z Administration of imatinib after allogeneic hematopoietic stem cell transplantation may improve disease-free survival for patients with Philadelphia chromosome-positive acute lymphoblastic leukemia J Hematol Oncol 2012 6 29 10.1186/1756-8722-5-29 22682059 PMC3407007

[B39] Matthes-Martin S Feuchtinger T Shaw PJ Engelhard D Hirsch HH Cordonnier C Ljungman P European guidelines for diagnosis and treatment of adenovirus infection in leukemia and stem cell transplantation: summary of ECIL-4 (2011) Transpl Infect Dis 2012 6 6 555 563 10.1111/tid.12022 23146063

[B40] Ison MG Respiratory viral infections in transplant recipients Antivir Ther 2007 6 4 Pt B 627 638 17944270

[B41] Leland DS Ginocchio CC Role of cell culture for virus detection in the age of technology Clin Microbiol Rev 2007 6 1 49 78 10.1128/CMR.00002-06 17223623 PMC1797634

[B42] Talbot HK Falsey AR The diagnosis of viral respiratory disease in older adults Clin Infect Dis 2010 6 5 747 751 20121411 10.1086/650486 PMC2826599

[B43] Hardie DR Korsman SN Hsiao NY Cytomegalovirus load in whole blood is more reliable for predicting and assessing CMV disease than pp 65 antigenaemia J Virol Methods 2013 6 1 166 168 10.1016/j.jviromet.2013.06.019 23792685

[B44] Yoshikawa T Significance of human herpesviruses to transplant recipients Curr Opin Infect Dis 2003 6 6 601 606 10.1097/00001432-200312000-00014 14624112

[B45] Yakushiji K Gondo H Kamezaki K Shigematsu K Hayashi S Kuroiwa M Taniguchi S Ohno Y Takase K Numata A Monitoring of cytomegalovirus reactivation after allogeneic stem cell transplantation: comparison of an antigenemia assay and quantitative real-time polymerase chain reaction Bone Marrow Transplant 2002 6 7 599 606 10.1038/sj.bmt.1703513 11979310

[B46] Li H Dummer JS Estes WR Meng S Wright PF Tang YW Measurement of human cytomegalovirus loads by quantitative real-time PCR for monitoring clinical intervention in transplant recipients J Clin Microbiol 2003 6 1 187 191 10.1128/JCM.41.1.187-191.2003 12517846 PMC149564

[B47] Fan H Robetorye RS Epstein-Barr Virus (EBV) load determination using real-time quantitative polymerase chain reaction Methods Mol Biol 2013 6 231 243 10.1007/978-1-62703-357-2_17 23666703

[B48] Makol A Kosuri K Tamkus D de Calaca MW Chang HT Lymphomatoid granulomatosis masquerading as interstitial pneumonia in a 66-year-old man: a case report and review of literature J Hematol Oncol 2009 6 39 10.1186/1756-8722-2-39 19732432 PMC2741488

[B49] Rooney CM Smith CA Ng CY Loftin SK Sixbey JW Gan Y Srivastava DK Bowman LC Krance RA Brenner MK Infusion of cytotoxic T cells for the prevention and treatment of Epstein-Barr virus-induced lymphoma in allogeneic transplant recipients Blood 1998 6 5 1549 1555 9716582

[B50] Leen AM Bollard CM Mendizabal AM Shpall EJ Szabolcs P Antin JH Kapoor N Pai SY Rowley SD Kebriaei P Multicenter study of banked third party virus-specific T-cells to treat severe viral infections after hematopoietic stem cell transplantation Blood 2013 6 26 5113 5123 10.1182/blood-2013-02-486324 23610374 PMC3695359

[B51] Einsele H Roosnek E Rufer N Sinzger C Riegler S Loffler J Grigoleit U Moris A Rammensee HG Kanz L Infusion of cytomegalovirus (CMV)-specific T cells for the treatment of CMV infection not responding to antiviral chemotherapy Blood 2002 6 11 3916 3922 10.1182/blood.V99.11.3916 12010789

[B52] Brion A Cahn JY Mougin C Angonin R Flesch M Deschaseaux ML Plouvier E Deconinck E Voillat L Racadot E Herpes virus-related lymphoproliferative disorders following allogeneic bone marrow transplantation: clinical and biological characteristics of six cases Nouv Rev Fr Hematol 1995 6 6 289 296 8907621

[B53] Erard V Chien JW Kim HW Nichols WG Flowers ME Martin PJ Corey L Boeckh M Airflow decline after myeloablative allogeneic hematopoietic cell transplantation: the role of community respiratory viruses J Infect Dis 2006 6 12 1619 1625 10.1086/504268 16703503 PMC7110078

[B54] Ghosh S Champlin RE Englund J Giralt SA Rolston K Raad I Jacobson K Neumann J Ippoliti C Mallik S Respiratory syncytial virus upper respiratory tract illnesses in adult blood and marrow transplant recipients: combination therapy with aerosolized ribavirin and intravenous immunoglobulin Bone Marrow Transplant 2000 6 7 751 755 10.1038/sj.bmt.1702228 10745261

[B55] Lewinsohn DM Bowden RA Mattson D Crawford SW Phase I study of intravenous ribavirin treatment of respiratory syncytial virus pneumonia after marrow transplantation Antimicrob Agents Chemother 1996 6 11 2555 2557 8913463 10.1128/aac.40.11.2555 PMC163574

[B56] Saral R Burns WH Laskin OL Santos GW Lietman PS Acyclovir prophylaxis of herpes-simplex-virus infections N Engl J Med 1981 6 2 63 67 10.1056/NEJM198107093050202 6264292

[B57] Ljungman P Cordonnier C Einsele H Englund J Machado CM Storek J Small T Vaccination of hematopoietic cell transplant recipients Bone Marrow Transplant 2009 6 8 521 526 10.1038/bmt.2009.263 19861986

[B58] Cheuk DK Chiang AK Lee TL Chan GC Ha SY Vaccines for prophylaxis of viral infections in patients with hematological malignancies Cochrane Database Syst Rev 2011 6 D6505 10.1002/14651858.CD006505.pub2 21412895

[B59] Engelhard D Mohty B de la Camara R Cordonnier C Ljungman P European guidelines for prevention and management of influenza in hematopoietic stem cell transplantation and leukemia patients: summary of ECIL-4 (2011), on behalf of ECIL, a joint venture of EBMT, EORTC, ICHS and ELN Transpl Infect Dis 2013 6 3 219 232 10.1111/tid.12054 23363310

[B60] Ruuskanen O Lahti E Jennings LC Murdoch DR Viral pneumonia Lancet 2011 6 9773 1264 1275 10.1016/S0140-6736(10)61459-6 21435708 PMC7138033

[B61] Barton TD Blumberg EA Viral pneumonias other than cytomegalovirus in transplant recipients Clin Chest Med 2005 6 4 707 720 10.1016/j.ccm.2005.06.004 16263407 PMC7119197

[B62] Ison MG Hayden FG Viral infections in immunocompromised patients: what's new with respiratory viruses? Curr Opin Infect Dis 2002 6 4 355 367 10.1097/00001432-200208000-00002 12130931

[B63] Symeonidis N Jakubowski A Pierre-Louis S Jaffe D Pamer E Sepkowitz K O'Reilly RJ Papanicolaou GA Invasive adenoviral infections in T-cell-depleted allogeneic hematopoietic stem cell transplantation: high mortality in the era of cidofovir Transpl Infect Dis 2007 6 2 108 113 10.1111/j.1399-3062.2006.00184.x 17461995

[B64] Ljungman P Ward KN Crooks BN Parker A Martino R Shaw PJ Brinch L Brune M De La Camara R Dekker A Respiratory virus infections after stem cell transplantation: a prospective study from the Infectious Diseases Working Party of the European Group for Blood and Marrow Transplantation Bone Marrow Transplant 2001 6 5 479 484 10.1038/sj.bmt.1703139 11593321

[B65] Travi G Pergam SA Cytomegalovirus pneumonia in hematopoietic stem cell recipients J Intensive Care Med 2013 6 23753231 10.1177/0885066613476454 PMC3775875 23753231

[B66] Granena A Carreras E Rozman C Salgado C Sierra J Algara M Rovira M Valls A Interstitial pneumonitis after BMT: 15 years experience in a single institution Bone Marrow Transplant 1993 6 6 453 458 8392885

[B67] Boeckh M Leisenring W Riddell SR Bowden RA Huang ML Myerson D Stevens-Ayers T Flowers ME Cunningham T Corey L Late cytomegalovirus disease and mortality in recipients of allogeneic hematopoietic stem cell transplants: importance of viral load and T-cell immunity Blood 2003 6 2 407 414 10.1182/blood-2002-03-0993 12393659

[B68] van Esser JW van der Holt B Meijer E Niesters HG Trenschel R Thijsen SF van Loon AM Frassoni F Bacigalupo A Schaefer UW Epstein-Barr virus (EBV) reactivation is a frequent event after allogeneic stem cell transplantation (SCT) and quantitatively predicts EBV-lymphoproliferative disease following T-cell–depleted SCT Blood 2001 6 4 972 978 10.1182/blood.V98.4.972 11493441

[B69] Schmidt-Hieber M Schwender J Heinz WJ Zabelina T Kuhl JS Mousset S Schuttrumpf S Junghanss C Silling G Basara N Viral encephalitis after allogeneic stem cell transplantation: a rare complication with distinct characteristics of different causative agents Haematologica 2011 6 1 142 149 10.3324/haematol.2010.029876 20851868 PMC3012778

[B70] Wu M Huang F Jiang X Fan Z Zhou H Liu C Jiang Q Zhang Y Zhao K Xuan L Herpesvirus-associated central nervous system diseases after allogeneic hematopoietic stem cell transplantation PLoS One 2013 6 10 e77805 10.1371/journal.pone.0077805 24124621 PMC3790760

[B71] Schmidt-Hieber M Zweigner J Uharek L Blau IW Thiel E Central nervous system infections in immunocompromised patients: update on diagnostics and therapy Leuk Lymphoma 2009 6 1 24 36 10.1080/10428190802517740 19031169

[B72] Walter JE Mitchell DK Astrovirus infection in children Curr Opin Infect Dis 2003 6 3 247 253 10.1097/00001432-200306000-00011 12821816

[B73] van Kraaij MG Dekker AW Verdonck LF van Loon AM Vinje J Koopmans MP Rozenberg-Arska M Infectious gastro-enteritis: an uncommon cause of diarrhoea in adult allogeneic and autologous stem cell transplant recipients Bone Marrow Transplant 2000 6 3 299 303 10.1038/sj.bmt.1702484 10967569 PMC7091909

[B74] Anderson EJ Weber SG Rotavirus infection in adults Lancet Infect Dis 2004 6 2 91 99 10.1016/S1473-3099(04)00928-4 14871633 PMC7106507

[B75] Yusuf U Hale GA Carr J Gu Z Benaim E Woodard P Kasow KA Horwitz EM Leung W Srivastava DK Cidofovir for the treatment of adenoviral infection in pediatric hematopoietic stem cell transplant patients Transplantation 2006 6 10 1398 1404 10.1097/01.tp.0000209195.95115.8e 16732176

[B76] Ljungman P Ribaud P Eyrich M Matthes-Martin S Einsele H Bleakley M Machaczka M Bierings M Bosi A Gratecos N Cidofovir for adenovirus infections after allogeneic hematopoietic stem cell transplantation: a survey by the Infectious Diseases Working Party of the European Group for Blood and Marrow Transplantation Bone Marrow Transplant 2003 6 6 481 486 10.1038/sj.bmt.1703798 12665844

[B77] Robles JD Cheuk DK Ha SY Chiang AK Chan GC Norovirus infection in pediatric hematopoietic stem cell transplantation recipients: incidence, risk factors, and outcome Biol Blood Marrow Transplant 2012 6 12 1883 1889 10.1016/j.bbmt.2012.07.005 22796532

[B78] van Burik JA Lawatsch EJ DeFor TE Weisdorf DJ Cytomegalovirus enteritis among hematopoietic stem cell transplant recipients Biol Blood Marrow Transplant 2001 6 12 674 679 10.1053/bbmt.2001.v7.pm11787530 11787530

[B79] Locasciulli A Testa M Valsecchi MG Bacigalupo A Solinas S Tomas JF Ljungman P Alberti A The role of hepatitis C and B virus infections as risk factors for severe liver complications following allogeneic BMT: a prospective study by the Infectious Disease Working Party of the European Blood and Marrow Transplantation Group Transplantation 1999 6 10 1486 1491 10.1097/00007890-199911270-00010 10589944

[B80] Lalazar G Rund D Shouval D Screening, prevention and treatment of viral hepatitis B reactivation in patients with haematological malignancies Br J Haematol 2007 6 5 699 712 10.1111/j.1365-2141.2006.06465.x 17338776

[B81] Locasciulli A Bruno B Alessandrino EP Meloni G Arcese W Bandini G Cassibba V Rotoli B Morra E Majolino I Hepatitis reactivation and liver failure in haemopoietic stem cell transplants for hepatitis B virus (HBV)/hepatitis C virus (HCV) positive recipients: a retrospective study by the Italian group for blood and marrow transplantation Bone Marrow Transplant 2003 6 4 295 300 10.1038/sj.bmt.1703826 12621466

[B82] Cui Y Jia J Update on epidemiology of hepatitis B and C in China J Gastroenterol Hepatol 2013 6 Suppl 1 7 10 23855289 10.1111/jgh.12220

[B83] Strasser SI Myerson D Spurgeon CL Sullivan KM Storer B Schoch HG Kim S Flowers ME McDonald GB Hepatitis C virus infection and bone marrow transplantation: a cohort study with 10-year follow-up Hepatology 1999 6 6 1893 1899 10.1002/hep.510290609 10347135

[B84] Tsuboi K Kishi K Ohmachi K Yasuda Y Shimizu T Inoue H Matsumoto M Hattori K Yoshiba F Watanabe S Multivariate analysis of risk factors for hemorrhagic cystitis after hematopoietic stem cell transplantation Bone Marrow Transplant 2003 6 9 903 907 10.1038/sj.bmt.1704240 14561991

[B85] Kloos RQ Boelens JJ de Jong TP Versluys B Bierings M Hemorrhagic cystitis in a cohort of pediatric transplantations: incidence, treatment, outcome, and risk factors Biol Blood Marrow Transplant 2013 6 8 1263 1266 10.1016/j.bbmt.2013.05.014 23711594

[B86] Bedi A Miller CB Hanson JL Goodman S Ambinder RF Charache P Arthur RR Jones RJ Association of BK virus with failure of prophylaxis against hemorrhagic cystitis following bone marrow transplantation J Clin Oncol 1995 6 5 1103 1109 7738616 10.1200/JCO.1995.13.5.1103

[B87] Leung AY Mak R Lie AK Yuen KY Cheng VC Liang R Kwong YL Clinicopathological features and risk factors of clinically overt haemorrhagic cystitis complicating bone marrow transplantation Bone Marrow Transplant 2002 6 6 509 513 10.1038/sj.bmt.1703415 11960271

[B88] Han TT Xu LP Liu DH Liu KY Fu HX Zhao XY Zhao XS Huang XJ Cytomegalovirus is a potential risk factor for late-onset hemorrhagic cystitis following allogeneic hematopoietic stem cell transplantation Am J Hematol 2013 doi: 10.1002/ajh.23584 10.1002/ajh.23584 24009106

[B89] Akiyama H Kurosu T Sakashita C Inoue T Mori S Ohashi K Tanikawa S Sakamaki H Onozawa Y Chen Q Adenovirus is a key pathogen in hemorrhagic cystitis associated with bone marrow transplantation Clin Infect Dis 2001 6 9 1325 1330 10.1086/319992 11303268

[B90] Tsao SY Chang KC Chen YP Yeh YM Su WC Chen TY Cytomegalovirus and Epstein-Barr virus-associated post-transplant lymphoproliferative disorder after allogeneic hematopoietic stem cell transplantation Ann Hematol 2011 6 1 113 114 10.1007/s00277-010-0947-8 20369358

[B91] Manez R Breinig MC Linden P Wilson J Torre-Cisneros J Kusne S Dummer S Ho M Posttransplant lymphoproliferative disease in primary Epstein-Barr virus infection after liver transplantation: the role of cytomegalovirus disease J Infect Dis 1997 6 6 1462 1467 10.1086/514142 9395355

[B92] Comoli P Basso S Zecca M Pagliara D Baldanti F Bernardo ME Barberi W Moretta A Labirio M Paulli M Preemptive therapy of EBV-related lymphoproliferative disease after pediatric haploidentical stem cell transplantation Am J Transplant 2007 6 6 1648 1655 10.1111/j.1600-6143.2007.01823.x 17511690

[B93] Zutter MM Martin PJ Sale GE Shulman HM Fisher L Thomas ED Durnam DM Epstein-Barr virus lymphoproliferation after bone marrow transplantation Blood 1988 6 2 520 529 2840986

[B94] Sundin M Le Blanc K Ringden O Barkholt L Omazic B Lergin C Levitsky V Remberger M The role of HLA mismatch, splenectomy and recipient Epstein-Barr virus seronegativity as risk factors in post-transplant lymphoproliferative disorder following allogeneic hematopoietic stem cell transplantation Haematologica 2006 6 8 1059 1067 16885046

[B95] Curtis RE Travis LB Rowlings PA Socie G Kingma DW Banks PM Jaffe ES Sale GE Horowitz MM Witherspoon RP Risk of lymphoproliferative disorders after bone marrow transplantation: a multi-institutional study Blood 1999 6 7 2208 2216 10498590

[B96] Meijer E Cornelissen JJ Epstein-Barr virus-associated lymphoproliferative disease after allogeneic haematopoietic stem cell transplantation: molecular monitoring and early treatment of high-risk patients Curr Opin Hematol 2008 6 6 576 585 10.1097/MOH.0b013e328311f438 18832928

[B97] Chiang KY Hazlett LJ Godder KT Abhyankar SH Christiansen NP van Rhee F Lee CG Bridges K Parrish RS Henslee-Downey PJ Epstein-Barr virus-associated B cell lymphoproliferative disorder following mismatched related T cell-depleted bone marrow transplantation Bone Marrow Transplant 2001 6 12 1117 1123 10.1038/sj.bmt.1703311 11803352

[B98] Nozzoli C Bartolozzi B Guidi S Orsi A Vannucchi AM Leoni F Bosi A Epstein-Barr virus-associated post-transplant lymphoproliferative disease with central nervous system involvement after unrelated allogeneic hematopoietic stem cell transplantation Leuk Lymphoma 2006 6 1 167 169 10.1080/10428190500254208 16321845

[B99] Shimizu H Saitoh T Koya H Yuzuriha A Hoshino T Hatsumi N Takada S Nagaki T Nojima Y Sakura T Discrepancy in EBV-DNA load between peripheral blood and cerebrospinal fluid in a patient with isolated CNS post-transplant lymphoproliferative disorder Int J Hematol 2011 6 5 495 498 10.1007/s12185-011-0951-3 22038015

[B100] Kittan NA Beier F Kurz K Niller HH Egger L Jilg W Andreesen R Holler E Hildebrandt GC Isolated cerebral manifestation of Epstein-Barr virus-associated post-transplant lymphoproliferative disorder after allogeneic hematopoietic stem cell transplantation: a case of clinical and diagnostic challenges Transpl Infect Dis 2011 6 5 524 530 10.1111/j.1399-3062.2011.00621.x 21395956

[B101] DiNardo CD Tsai DE Treatment advances in posttransplant lymphoproliferative disease Curr Opin Hematol 2010 6 4 368 374 10.1097/MOH.0b013e328339018c 20473161

[B102] Ljungman P Griffiths P Paya C Definitions of cytomegalovirus infection and disease in transplant recipients Clin Infect Dis 2002 6 8 1094 1097 10.1086/339329 11914998

[B103] Ljungman P Hakki M Boeckh M Cytomegalovirus in hematopoietic stem cell transplant recipients Infect Dis Clin North Am 2010 6 2 319 337 10.1016/j.idc.2010.01.008 20466273

[B104] Meyers JD Flournoy N Thomas ED Infection with herpes simplex virus and cell-mediated immunity after marrow transplant J Infect Dis 1980 6 3 338 346 10.1093/infdis/142.3.338 6255035

[B105] Kawamura K Wada H Yamasaki R Ishihara Y Sakamoto K Ashizawa M Sato M Machishima T Terasako K Kimura SI Low-dose acyclovir prophylaxis for the prevention of herpes simplex virus disease after allogeneic hematopoietic stem cell transplantation Transpl Infect Dis 2013 6 5 457 465 23895431 10.1111/tid.12118

[B106] Dykewicz CA Kaplan JE Guidelines for preventing opportunistic infections among hematopoietic stem cell transplant recipients MMWR Recomm Rep 2000 6 RR-10 1 125 E1-E7 11718124

[B107] Liesveld JL Abboud CN Ifthikharuddin JJ Lancet JE Wedow LA Oliva J Stamm CG Nichols D Oral valacyclovir versus intravenous acyclovir in preventing herpes simplex virus infections in autologous stem cell transplant recipients Biol Blood Marrow Transplant 2002 6 12 662 665 10.1053/bbmt.2002.v8.abbmt080662 12523578

[B108] Orlowski RZ Mills SR Hartley EE Ye X Piantadosi S Ambinder RF Gore SD Miller CB Oral valacyclovir as prophylaxis against herpes simplex virus reactivation during high dose chemotherapy for leukemia Leuk Lymphoma 2004 6 11 2215 2219 10.1080/10428190410001733763 15512809

[B109] Hoglund M Ljungman P Weller S Comparable aciclovir exposures produced by oral valaciclovir and intravenous aciclovir in immunocompromised cancer patients J Antimicrob Chemother 2001 6 6 855 861 10.1093/jac/47.6.855 11389118

[B110] Blot N Schneider P Young P Janvresse C Dehesdin D Tron P Vannier JP Treatment of an acyclovir and foscarnet-resistant herpes simplex virus infection with cidofovir in a child after an unrelated bone marrow transplant Bone Marrow Transplant 2000 6 8 903 905 10.1038/sj.bmt.1702591 11081393

[B111] Zaia JA Levin MJ Preblud SR Leszczynski J Wright GG Ellis RJ Curtis AC Valerio MA LeGore J Evaluation of varicella-zoster immune globulin: protection of immunosuppressed children after household exposure to varicella J Infect Dis 1983 6 4 737 743 10.1093/infdis/147.4.737 6341478

[B112] Feldman S Lott L Varicella in children with cancer: impact of antiviral therapy and prophylaxis Pediatrics 1987 6 4 465 472 2821476

[B113] Weinstock DM Boeckh M Sepkowitz KA Postexposure prophylaxis against varicella zoster virus infection among hematopoietic stem cell transplant recipients Biol Blood Marrow Transplant 2006 6 10 1096 1097 10.1016/j.bbmt.2006.06.005 17084374

[B114] Ljungman P Wilczek H Gahrton G Gustavsson A Lundgren G Lonnqvist B Ringden O Wahren B Long-term acyclovir prophylaxis in bone marrow transplant recipients and lymphocyte proliferation responses to herpes virus antigens in vitro Bone Marrow Transplant 1986 6 2 185 192 2844333

[B115] Perren TJ Powles RL Easton D Stolle K Selby PJ Prevention of herpes zoster in patients by long-term oral acyclovir after allogeneic bone marrow transplantation Am J Med 1988 6 2A 99 101 3044103

[B116] Boeckh M Kim HW Flowers ME Meyers JD Bowden RA Long-term acyclovir for prevention of varicella zoster virus disease after allogeneic hematopoietic cell transplantation–a randomized double-blind placebo-controlled study Blood 2006 6 5 1800 1805 10.1182/blood-2005-09-3624 16282339 PMC1895699

[B117] Oshima K Takahashi T Mori T Matsuyama T Usuki K Asano-Mori Y Nakahara F Okamoto S Kurokawa M Kanda Y One-year low-dose valacyclovir as prophylaxis for varicella zoster virus disease after allogeneic hematopoietic stem cell transplantation. A prospective study of the Japan Hematology and Oncology Clinical Study Group Transpl Infect Dis 2010 6 5 421 427 10.1111/j.1399-3062.2010.00541.x 20626711

[B118] Serota FT Starr SE Bryan CK Koch PA Plotkin SA August CS Acyclovir treatment of herpes zoster infections. Use in children undergoing bone marrow transplantation JAMA 1982 6 15 2132 2135 10.1001/jama.1982.03320400044032 7038177

[B119] Shepp DH Dandliker PS Meyers JD Treatment of varicella-zoster virus infection in severely immunocompromised patients. A randomized comparison of acyclovir and vidarabine N Engl J Med 1986 6 4 208 212 10.1056/NEJM198601233140404 3001523

[B120] Tyring S Belanger R Bezwoda W Ljungman P Boon R Saltzman RL A randomized, double-blind trial of famciclovir versus acyclovir for the treatment of localized dermatomal herpes zoster in immunocompromised patients Cancer Invest 2001 6 1 13 22 10.1081/CNV-100000070 11291551

[B121] Wassilew SW Wutzler P Oral brivudin in comparison with acyclovir for improved therapy of herpes zoster in immunocompetent patients: results of a randomized, double-blind, multicentered study Antiviral Res 2003 6 1 49 56 10.1016/S0166-3542(03)00065-2 12834860

[B122] Reusser P Cordonnier C Einsele H Engelhard D Link D Locasciulli A Ljungman P European survey of herpesvirus resistance to antiviral drugs in bone marrow transplant recipients. Infectious Diseases Working Party of the European Group for Blood and Marrow Transplantation (EBMT) Bone Marrow Transplant 1996 6 5 813 817 8733703

[B123] Yanada M Yamamoto K Emi N Naoe T Suzuki R Taji H Iida H Shimokawa T Kohno A Mizuta S Cytomegalovirus antigenemia and outcome of patients treated with pre-emptive ganciclovir: retrospective analysis of 241 consecutive patients undergoing allogeneic hematopoietic stem cell transplantation Bone Marrow Transplant 2003 6 8 801 807 10.1038/sj.bmt.1704232 14520425

[B124] Ljungman P Engelhard D Link H Biron P Brandt L Brunet S Cordonnier C Debusscher L de Laurenzi A Kolb HJ Treatment of interstitial pneumonitis due to cytomegalovirus with ganciclovir and intravenous immune globulin: experience of European Bone Marrow Transplant Group Clin Infect Dis 1992 6 4 831 835 10.1093/clinids/14.4.831 1315585

[B125] Nichols WG Corey L Gooley T Drew WL Miner R Huang M Davis C Boeckh M Rising pp 65 antigenemia during preemptive anticytomegalovirus therapy after allogeneic hematopoietic stem cell transplantation: risk factors, correlation with DNA load, and outcomes Blood 2001 6 4 867 874 10.1182/blood.V97.4.867 11159510

[B126] Einsele H Ehninger G Hebart H Wittkowski KM Schuler U Jahn G Mackes P Herter M Klingebiel T Loffler J Polymerase chain reaction monitoring reduces the incidence of cytomegalovirus disease and the duration and side effects of antiviral therapy after bone marrow transplantation Blood 1995 6 7 2815 2820 7670117

[B127] Winston DJ Ho WG Bartoni K Du Mond C Ebeling DF Buhles WC Champlin RE Ganciclovir prophylaxis of cytomegalovirus infection and disease in allogeneic bone marrow transplant recipients. Results of a placebo-controlled, double-blind trial Ann Intern Med 1993 6 3 179 184 10.7326/0003-4819-118-3-199302010-00004 8380243

[B128] Goodrich JM Bowden RA Fisher L Keller C Schoch G Meyers JD Ganciclovir prophylaxis to prevent cytomegalovirus disease after allogeneic marrow transplant Ann Intern Med 1993 6 3 173 178 10.7326/0003-4819-118-3-199302010-00003 8380242

[B129] Einsele H Reusser P Bornhauser M Kalhs P Ehninger G Hebart H Chalandon Y Kroger N Hertenstein B Rohde F Oral valganciclovir leads to higher exposure to ganciclovir than intravenous ganciclovir in patients following allogeneic stem cell transplantation Blood 2006 6 7 3002 3008 10.1182/blood-2005-09-3786 16352807

[B130] Cooper N Rao K Goulden N Webb D Amrolia P Veys P The use of reduced-intensity stem cell transplantation in haemophagocytic lymphohistiocytosis and Langerhans cell histiocytosis Bone Marrow Transplant 2008 6 Suppl 2 S47 S50 18978744 10.1038/bmt.2008.283

[B131] van der Heiden PL Kalpoe JS Barge RM Willemze R Kroes AC Schippers EF Oral valganciclovir as pre-emptive therapy has similar efficacy on cytomegalovirus DNA load reduction as intravenous ganciclovir in allogeneic stem cell transplantation recipients Bone Marrow Transplant 2006 6 7 693 698 10.1038/sj.bmt.1705311 16501590

[B132] Reed EC Bowden RA Dandliker PS Lilleby KE Meyers JD Treatment of cytomegalovirus pneumonia with ganciclovir and intravenous cytomegalovirus immunoglobulin in patients with bone marrow transplants Ann Intern Med 1988 6 10 783 788 10.7326/0003-4819-109-10-783 2847610

[B133] Ljungman P Deliliers GL Platzbecker U Matthes-Martin S Bacigalupo A Einsele H Ullmann J Musso M Trenschel R Ribaud P Cidofovir for cytomegalovirus infection and disease in allogeneic stem cell transplant recipients. The Infectious Diseases Working Party of the European Group for Blood and Marrow Transplantation Blood 2001 6 2 388 392 10.1182/blood.V97.2.388 11154213

[B134] Blyth E Clancy L Simms R Ma CK Burgess J Deo S Byth K Dubosq MC Shaw PJ Micklethwaite KP Donor-derived CMV-specific T cells reduce the requirement for CMV-directed pharmacotherapy after allogeneic stem cell transplantation Blood 2013 6 18 3745 3758 10.1182/blood-2012-08-448977 23435462

[B135] Liu Q Xuan L Liu H Huang F Zhou H Fan Z Zhao K Wu M Xu L Zhai X Molecular monitoring and stepwise preemptive therapy for Epstein-Barr virus viremia after allogeneic stem cell transplantation Am J Hematol 2013 6 7 550 555 10.1002/ajh.23452 23564232

[B136] Stevens SJ Verschuuren EA Pronk I van Der Bij W Harmsen MC The TH Meijer CJ van Den Brule AJ Middeldorp JM Frequent monitoring of Epstein-Barr virus DNA load in unfractionated whole blood is essential for early detection of posttransplant lymphoproliferative disease in high-risk patients Blood 2001 6 5 1165 1171 10.1182/blood.V97.5.1165 11222357

[B137] Heslop HE Slobod KS Pule MA Hale GA Rousseau A Smith CA Bollard CM Liu H Wu MF Rochester RJ Long-term outcome of EBV-specific T-cell infusions to prevent or treat EBV-related lymphoproliferative disease in transplant recipients Blood 2010 6 5 925 935 10.1182/blood-2009-08-239186 19880495 PMC2817637

[B138] Peric Z Cahu X Chevallier P Brissot E Malard F Guillaume T Delaunay J Ayari S Dubruille V Le Gouill S Features of Epstein-Barr Virus (EBV) reactivation after reduced intensity conditioning allogeneic hematopoietic stem cell transplantation Leukemia 2011 6 6 932 938 10.1038/leu.2011.26 21350556

[B139] Perrine SP Hermine O Small T Suarez F O'Reilly R Boulad F Fingeroth J Askin M Levy A Mentzer SJ A phase 1/2 trial of arginine butyrate and ganciclovir in patients with Epstein-Barr virus-associated lymphoid malignancies Blood 2007 6 6 2571 2578 10.1182/blood-2006-01-024703 17119113 PMC1852196

[B140] Faller DV Mentzer SJ Perrine SP Induction of the Epstein-Barr virus thymidine kinase gene with concomitant nucleoside antivirals as a therapeutic strategy for Epstein-Barr virus-associated malignancies Curr Opin Oncol 2001 6 5 360 367 10.1097/00001622-200109000-00008 11555713

[B141] Elstrom RL Andreadis C Aqui NA Ahya VN Bloom RD Brozena SC Olthoff KM Schuster SJ Nasta SD Stadtmauer EA Treatment of PTLD with rituximab or chemotherapy Am J Transplant 2006 6 3 569 576 10.1111/j.1600-6143.2005.01211.x 16468968

[B142] Choquet S Leblond V Herbrecht R Socie G Stoppa AM Vandenberghe P Fischer A Morschhauser F Salles G Feremans W Efficacy and safety of rituximab in B-cell post-transplantation lymphoproliferative disorders: results of a prospective multicenter phase 2 study Blood 2006 6 8 3053 3057 10.1182/blood-2005-01-0377 16254143

[B143] Milpied N Vasseur B Parquet N Garnier JL Antoine C Quartier P Carret AS Bouscary D Faye A Bourbigot B Humanized anti-CD20 monoclonal antibody (Rituximab) in post transplant B-lymphoproliferative disorder: a retrospective analysis on 32 patients Ann Oncol 2000 6 Suppl 1 113 116 10.1093/annonc/11.suppl_1.S113 10707791

[B144] Faye A Quartier P Reguerre Y Lutz P Carret AS Dehee A Rohrlich P Peuchmaur M Matthieu-Boue A Fischer A Chimaeric anti-CD20 monoclonal antibody (rituximab) in post-transplant B-lymphoproliferative disorder following stem cell transplantation in children Br J Haematol 2001 6 1 112 118 10.1046/j.1365-2141.2001.03041.x 11722420

[B145] Styczynski J Einsele H Gil L Ljungman P Outcome of treatment of Epstein-Barr virus-related post-transplant lymphoproliferative disorder in hematopoietic stem cell recipients: a comprehensive review of reported cases Transpl Infect Dis 2009 6 5 383 392 10.1111/j.1399-3062.2009.00411.x 19558376

[B146] Wagner HJ Cheng YC Huls MH Gee AP Kuehnle I Krance RA Brenner MK Rooney CM Heslop HE Prompt versus preemptive intervention for EBV lymphoproliferative disease Blood 2004 6 10 3979 3981 10.1182/blood-2003-12-4287 14751931

[B147] Gross TG Bucuvalas JC Park JR Greiner TC Hinrich SH Kaufman SS Langnas AN McDonald RA Ryckman FC Shaw BW Low-dose chemotherapy for Epstein-Barr virus-positive post-transplantation lymphoproliferative disease in children after solid organ transplantation J Clin Oncol 2005 6 27 6481 6488 10.1200/JCO.2005.08.074 16170157

[B148] Trappe R Oertel S Leblond V Mollee P Sender M Reinke P Neuhaus R Lehmkuhl H Horst HA Salles G Sequential treatment with rituximab followed by CHOP chemotherapy in adult B-cell post-transplant lymphoproliferative disorder (PTLD): the prospective international multicentre phase 2 PTLD-1 trial Lancet Oncol 2012 6 2 196 206 10.1016/S1470-2045(11)70300-X 22173060

[B149] Gong JZ Bayerl MG Sandhaus LM Sebastian S Rehder CW Routbort M Lagoo AS Szabolcs P Chiu J Comito M Posttransplant lymphoproliferative disorder after umbilical cord blood transplantation in children Am J Surg Pathol 2006 6 3 328 336 16538052 10.1097/01.pas.0000188030.63706.e7

[B150] Maximiano AC Sanchez RA Cantos SDIB Mendez GM Ronco IS Provencio PM Ocular relapse of primary brain lymphoma in immunocompetent patient, treated with intrathecal rituximab Clin Transl Oncol 2010 6 10 701 703 10.1007/s12094-010-0580-y 20947485

[B151] Bonney DK Htwe EE Turner A Kelsey A Shabani A Hughes S Hughes I Wynn RF Sustained response to intrathecal rituximab in EBV associated Post-transplant lymphoproliferative disease confined to the central nervous system following haematopoietic stem cell transplant Pediatr Blood Cancer 2012 6 3 459 461 10.1002/pbc.23134 21584931

[B152] Czyzewski K Styczynski J Krenska A Debski R Zajac-Spychala O Wachowiak J Wysocki M Intrathecal therapy with rituximab in central nervous system involvement of post-transplant lymphoproliferative disorder Leuk Lymphoma 2013 6 3 503 506 10.3109/10428194.2012.718342 22873830

[B153] Rubenstein JL Fridlyand J Abrey L Shen A Karch J Wang E Issa S Damon L Prados M McDermott M Phase I study of intraventricular administration of rituximab in patients with recurrent CNS and intraocular lymphoma J Clin Oncol 2007 6 11 1350 1356 10.1200/JCO.2006.09.7311 17312328

[B154] Kinch A Oberg G Arvidson J Falk KI Linde A Pauksens K Post-transplant lymphoproliferative disease and other Epstein-Barr virus diseases in allogeneic haematopoietic stem cell transplantation after introduction of monitoring of viral load by polymerase chain reaction Scand J Infect Dis 2007 6 3 235 244 10.1080/00365540600978906 17366054

[B155] Tokimasa S Hara J Osugi Y Ohta H Matsuda Y Fujisaki H Sawada A Kim JY Sashihara J Amou K Ganciclovir is effective for prophylaxis and treatment of human herpesvirus-6 in allogeneic stem cell transplantation Bone Marrow Transplant 2002 6 7 595 598 10.1038/sj.bmt.1703423 11979309

[B156] Rapaport D Engelhard D Tagger G Or R Frenkel N Antiviral prophylaxis may prevent human herpesvirus-6 reactivation in bone marrow transplant recipients Transpl Infect Dis 2002 6 1 10 16 10.1034/j.1399-3062.2002.040101.x 12123421

[B157] Zerr DM Gupta D Huang ML Carter R Corey L Effect of antivirals on human herpesvirus 6 replication in hematopoietic stem cell transplant recipients Clin Infect Dis 2002 6 3 309 317 10.1086/338044 11774077

[B158] Gantt S Casper C Human herpesvirus 8-associated neoplasms: the roles of viral replication and antiviral treatment Curr Opin Infect Dis 2011 6 4 295 301 10.1097/QCO.0b013e3283486d04 21666458 PMC4059200

[B159] Luppi M Barozzi P Schulz TF Setti G Staskus K Trovato R Narni F Donelli A Maiorana A Marasca R Bone marrow failure associated with human herpesvirus 8 infection after transplantation N Engl J Med 2000 6 19 1378 1385 10.1056/NEJM200011093431905 11070102

[B160] Luppi M Barozzi P Schulz TF Trovato R Donelli A Narni F Sheldon J Marasca R Torelli G Nonmalignant disease associated with human herpesvirus 8 reactivation in patients who have undergone autologous peripheral blood stem cell transplantation Blood 2000 6 7 2355 2357 11001882

[B161] Erer B Angelucci E Muretto P Ripalti M Rapa S Gaziev D Baronciani D Kaposi's sarcoma after allogeneic bone marrow transplantation Bone Marrow Transplant 1997 6 6 629 631 10.1038/sj.bmt.1700703 9085744

[B162] Cuzzola M Irrera G Iacopino O Cuzzocrea A Messina G Console G Iacopino P Morabito F Bone marrow failure associated with herpesvirus 8 infection in a patient undergoing autologous peripheral blood stem cell transplantation Clin Infect Dis 2003 6 7 e102 e106 10.1086/377268 13130419

[B163] Small TN Casson A Malak SF Boulad F Kiehn TE Stiles J Ushay HM Sepkowitz KA Respiratory syncytial virus infection following hematopoietic stem cell transplantation Bone Marrow Transplant 2002 6 4 321 327 10.1038/sj.bmt.1703365 11896429

[B164] Shah JN Chemaly RF Management of RSV infections in adult recipients of hematopoietic stem cell transplantation Blood 2011 6 10 2755 2763 10.1182/blood-2010-08-263400 21139081

[B165] Nichols WG Corey L Gooley T Davis C Boeckh M Parainfluenza virus infections after hematopoietic stem cell transplantation: risk factors, response to antiviral therapy, and effect on transplant outcome Blood 2001 6 3 573 578 10.1182/blood.V98.3.573 11468152

[B166] Elizaga J Olavarria E Apperley J Goldman J Ward K Parainfluenza virus 3 infection after stem cell transplant: relevance to outcome of rapid diagnosis and ribavirin treatment Clin Infect Dis 2001 6 3 413 418 10.1086/318498 11170949

[B167] Milano F Campbell AP Guthrie KA Kuypers J Englund JA Corey L Boeckh M Human rhinovirus and coronavirus detection among allogeneic hematopoietic stem cell transplantation recipients Blood 2010 6 10 2088 2094 10.1182/blood-2009-09-244152 20042728 PMC2837322

[B168] Tomblyn M Chiller T Einsele H Gress R Sepkowitz K Storek J Wingard JR Young JA Boeckh MJ Guidelines for preventing infectious complications among hematopoietic cell transplantation recipients: a global perspective Biol Blood Marrow Transplant 2009 6 10 1143 1238 10.1016/j.bbmt.2009.06.019 19747629 PMC3103296

[B169] Chemaly RF Ghosh S Bodey GP Rohatgi N Safdar A Keating MJ Champlin RE Aguilera EA Tarrand JJ Raad II Respiratory viral infections in adults with hematologic malignancies and human stem cell transplantation recipients: a retrospective study at a major cancer center Medicine (Baltimore) 2006 6 5 278 287 10.1097/01.md.0000232560.22098.4e 16974212

[B170] Boeckh M Englund J Li Y Miller C Cross A Fernandez H Kuypers J Kim H Gnann J Whitley R Randomized controlled multicenter trial of aerosolized ribavirin for respiratory syncytial virus upper respiratory tract infection in hematopoietic cell transplant recipients Clin Infect Dis 2007 6 2 245 249 10.1086/509930 17173225

[B171] DeVincenzo JP Hirsch RL Fuentes RJ Top FJ Respiratory syncytial virus immune globulin treatment of lower respiratory tract infection in pediatric patients undergoing bone marrow transplantation - a compassionate use experience Bone Marrow Transplant 2000 6 2 161 165 10.1038/sj.bmt.1702118 10673674

[B172] de Fontbrune FS Robin M Porcher R Scieux C de Latour RP Ferry C Rocha V Boudjedir K Devergie A Bergeron A Palivizumab treatment of respiratory syncytial virus infection after allogeneic hematopoietic stem cell transplantation Clin Infect Dis 2007 6 8 1019 1024 10.1086/521912 17879919

[B173] Boeckh M Berrey MM Bowden RA Crawford SW Balsley J Corey L Phase 1 evaluation of the respiratory syncytial virus-specific monoclonal antibody palivizumab in recipients of hematopoietic stem cell transplants J Infect Dis 2001 6 3 350 354 10.1086/322043 11443562

[B174] Vu D Peck AJ Nichols WG Varley C Englund JA Corey L Boeckh M Safety and tolerability of oseltamivir prophylaxis in hematopoietic stem cell transplant recipients: a retrospective case–control study Clin Infect Dis 2007 6 2 187 193 10.1086/518985 17578777

[B175] Englund JA Champlin RE Wyde PR Kantarjian H Atmar RL Tarrand J Yousuf H Regnery H Klimov AI Cox NJ Common emergence of amantadine- and rimantadine-resistant influenza A viruses in symptomatic immunocompromised adults Clin Infect Dis 1998 6 6 1418 1424 10.1086/516358 9636873

[B176] Klimov AI Rocha E Hayden FG Shult PA Roumillat LF Cox NJ Prolonged shedding of amantadine-resistant influenzae A viruses by immunodeficient patients: detection by polymerase chain reaction-restriction analysis J Infect Dis 1995 6 5 1352 1355 10.1093/infdis/172.5.1352 7594676

[B177] Johny AA Clark A Price N Carrington D Oakhill A Marks DI The use of zanamivir to treat influenza A and B infection after allogeneic stem cell transplantation Bone Marrow Transplant 2002 6 2 113 115 10.1038/sj.bmt.1703343 11850704

[B178] Fraaij PL van der Vries E Beersma MF Riezebos-Brilman A Niesters HG van der Eijk AA de Jong MD Reis MD Horrevorts AM Ridwan BU Evaluation of the antiviral response to zanamivir administered intravenously for treatment of critically ill patients with pandemic influenza A (H1N1) infection J Infect Dis 2011 6 5 777 782 10.1093/infdis/jir397 21844304 PMC3156108

[B179] Ljungman P de la Camara R Perez-Bercoff L Abecasis M Nieto CJ Cannata-Ortiz MJ Cordonnier C Einsele H Gonzalez-Vicent M Espigado I Outcome of pandemic H1N1 infections in hematopoietic stem cell transplant recipients Haematologica 2011 6 8 1231 1235 10.3324/haematol.2011.041913 21546495 PMC3148919

[B180] Williams JV Martino R Rabella N Otegui M Parody R Heck JM Crowe JJ A prospective study comparing human metapneumovirus with other respiratory viruses in adults with hematologic malignancies and respiratory tract infections J Infect Dis 2005 6 6 1061 1065 10.1086/432732 16107960 PMC1483060

[B181] La Rosa AM Champlin RE Mirza N Gajewski J Giralt S Rolston KV Raad I Jacobson K Kontoyiannis D Elting L Adenovirus infections in adult recipients of blood and marrow transplants Clin Infect Dis 2001 6 6 871 876 10.1086/319352 11247710

[B182] Bordigoni P Carret AS Venard V Witz F Le Faou A Treatment of adenovirus infections in patients undergoing allogeneic hematopoietic stem cell transplantation Clin Infect Dis 2001 6 9 1290 1297 10.1086/319984 11303263

[B183] Liles WC Cushing H Holt S Bryan C Hackman RC Severe adenoviral nephritis following bone marrow transplantation: successful treatment with intravenous ribavirin Bone Marrow Transplant 1993 6 4 409 412 8275042

[B184] Chakrabarti S Collingham KE Fegan CD Milligan DW Fulminant adenovirus hepatitis following unrelated bone marrow transplantation: failure of intravenous ribavirin therapy Bone Marrow Transplant 1999 6 11 1209 1211 10.1038/sj.bmt.1701788 10382964

[B185] Sivaprakasam P Carr TF Coussons M Khalid T Bailey AS Guiver M Mutton KJ Turner AJ Grainger JD Wynn RF Improved outcome from invasive adenovirus infection in pediatric patients after hemopoietic stem cell transplantation using intensive clinical surveillance and early intervention J Pediatr Hematol Oncol 2007 6 2 81 85 10.1097/MPH.0b013e318030875e 17279003

[B186] Feuchtinger T Matthes-Martin S Richard C Lion T Fuhrer M Hamprecht K Handgretinger R Peters C Schuster FR Beck R Safe adoptive transfer of virus-specific T-cell immunity for the treatment of systemic adenovirus infection after allogeneic stem cell transplantation Br J Haematol 2006 6 1 64 76 10.1111/j.1365-2141.2006.06108.x 16803570

[B187] Regn S Raffegerst S Chen X Schendel D Kolb HJ Roskrow M Ex vivo generation of cytotoxic T lymphocytes specific for one or two distinct viruses for the prophylaxis of patients receiving an allogeneic bone marrow transplant Bone Marrow Transplant 2001 6 1 53 64 10.1038/sj.bmt.1702752 11244438

[B188] Roddie C Paul JP Benjamin R Gallimore CI Xerry J Gray JJ Peggs KS Morris EC Thomson KJ Ward KN Allogeneic hematopoietic stem cell transplantation and norovirus gastroenteritis: a previously unrecognized cause of morbidity Clin Infect Dis 2009 6 7 1061 1068 10.1086/605557 19705974

[B189] Endo T Sakai T Fujimoto K Yamamoto S Takashima H Haseyama Y Nishio M Koizumi K Koike T Sawada K A possible role for lamivudine as prophylaxis against hepatitis B reactivation in carriers of hepatitis B who undergo chemotherapy and autologous peripheral blood stem cell transplantation for non-Hodgkin's lymphoma Bone Marrow Transplant 2001 6 4 433 436 10.1038/sj.bmt.1702804 11313673

[B190] Lau GK Liang R Wu PC Lee CK Lim WL Au WY Use of famciclovir to prevent HBV reactivation in HBsAg-positive recipients after allogeneic bone marrow transplantation J Hepatol 1998 6 3 359 368 10.1016/S0168-8278(98)80307-3 9551671

[B191] Ljungman P Andersson J Aschan J Bjorkstrand B Hagglund H Lonnqvist B Ringden O Winiarski J Oral ribavirin for prevention of severe liver disease caused by hepatitis C virus during allogeneic bone marrow transplantation Clin Infect Dis 1996 6 1 167 169 10.1093/clinids/23.1.167 8816147

[B192] Giardini C Galimberti M Lucarelli G Polchi P Angelucci E Baronciani D Erer B Gaziev D Piga A Di Gregorio F Alpha-interferon treatment of chronic hepatitis C after bone marrow transplantation for homozygous beta-thalassemia Bone Marrow Transplant 1997 6 9 767 772 10.1038/sj.bmt.1700968 9384479

[B193] Ljungman P Johansson N Aschan J Glaumann H Lonnqvist B Ringden O Sparrelid E Sonnerborg A Winiarski J Gahrton G Long-term effects of hepatitis C virus infection in allogeneic bone marrow transplant recipients Blood 1995 6 4 1614 1618 7632971

[B194] Balduzzi A Lucchini G Hirsch HH Basso S Cioni M Rovelli A Zincone A Grimaldi M Corti P Bonanomi S Polyomavirus JC-targeted T-cell therapy for progressive multiple leukoencephalopathy in a hematopoietic cell transplantation recipient Bone Marrow Transplant 2011 6 7 987 992 10.1038/bmt.2010.221 20921942

[B195] Leung AY Chan MT Yuen KY Cheng VC Chan KH Wong CL Liang R Lie AK Kwong YL Ciprofloxacin decreased polyoma BK virus load in patients who underwent allogeneic hematopoietic stem cell transplantation Clin Infect Dis 2005 6 4 528 537 10.1086/427291 15712075

[B196] Bridges B Donegan S Badros A Cidofovir bladder instillation for the treatment of BK hemorrhagic cystitis after allogeneic stem cell transplantation Am J Hematol 2006 6 7 535 537 10.1002/ajh.20567 16755571

[B197] Savona MR Newton D Frame D Levine JE Mineishi S Kaul DR Low-dose cidofovir treatment of BK virus-associated hemorrhagic cystitis in recipients of hematopoietic stem cell transplant Bone Marrow Transplant 2007 6 12 783 787 10.1038/sj.bmt.1705678 17438584

